# Spatio-Temporal Calibration of Multiple Kinect Cameras Using 3D Human Pose

**DOI:** 10.3390/s22228900

**Published:** 2022-11-17

**Authors:** Nadav Eichler, Hagit Hel-Or, Ilan Shimshoni

**Affiliations:** 1 Department of Computer Science, Haifa University, Haifa 3498838, Israel; 2 Department of Information Systems, Haifa University, Haifa 3498838, Israel

**Keywords:** multiple-camera setup, depth sensor, motion capture, 3D human pose estimation, extrinsic calibration, synchronization, Azure Kinect

## Abstract

RGB and depth cameras are extensively used for the 3D tracking of human pose and motion. Typically, these cameras calculate a set of 3D points representing the human body as a skeletal structure. The tracking capabilities of a single camera are often affected by noise and inaccuracies due to occluded body parts. Multiple-camera setups offer a solution to maximize coverage of the captured human body and to minimize occlusions. According to best practices, fusing information across multiple cameras typically requires spatio-temporal calibration. First, the cameras must synchronize their internal clocks. This is typically performed by physically connecting the cameras to each other using an external device or cable. Second, the pose of each camera relative to the other cameras must be calculated (Extrinsic Calibration). The state-of-the-art methods use specialized calibration session and devices such as a checkerboard to perform calibration. In this paper, we introduce an approach to the spatio-temporal calibration of multiple cameras which is designed to run on-the-fly without specialized devices or equipment requiring only the motion of the human body in the scene. As an example, the system is implemented and evaluated using Microsoft Azure Kinect. The study shows that the accuracy and robustness of this approach is on par with the state-of-the-art practices.

## 1. Introduction

**Figure 1:**
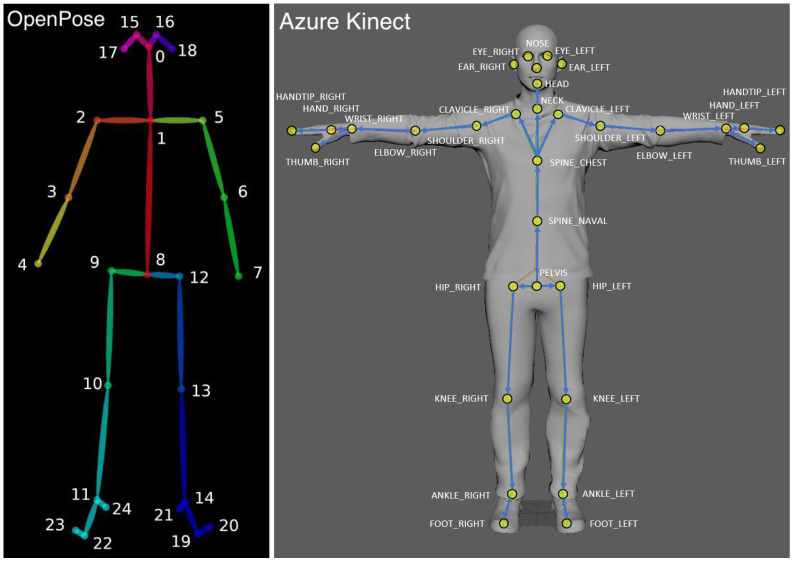
OpenPose (**Left**) and Azure Kinect (**Right**) skeletal joints maps.

RGB and depth cameras are extensively used for the tracking of human motion and extraction of body pose. Three-dimensional (3D) human pose estimation algorithms [1,2,3,4] are based on camera’s output streams (RGB, depth or both). The estimated pose is often given as a set of 3D points, representing the locations and orientations of the skeletal joints of the captured human body (see Figure 1). The tracking capabilities of a single camera often suffer due to the occlusion of body parts. Multiple-camera setups offer a solution to increase the coverage of the captured human body and to minimize the occlusions. However, the data acquired from multiple cameras must be merged in order to obtain coherent body tracking.

Thus, when using multiple cameras, two main requirements must be fulfilled in order to fuse the data across cameras:

1 Camera Synchronization (alignment between the cameras’ clocks).
2 Multi-Camera Calibration (calculating the mapping between cameras’ coordinate systems).

Traditional approaches to the synchronization and calibration of multiple cameras typically involve designated calibration sessions that require specialized tools such as light probes or checkerboards. Furthermore, the calibration sessions must be repeatedly performed to ensure calibration following camera shifts and drifts.

The goal of this article is to provide an alternative to the standard synchronization and calibration techniques. We present a simple, easy to use method even for non-technical savvy individuals. It relies on analyzing asynchronous 3D human pose records rather than performing the traditional synchronization and calibration sessions. The approach and methods presented are designed to fuse multiple camera recordings with minimum technical constraints, requiring only a moving body in the scene. The approach allows for a setup-and-go system that can continuously calibrate and can be operated by users with low technical skills, while maintaining the state-of-the-art practices in terms of accuracy. It can be performed continuously on-the-fly during recording or even retrospectively. Thus, calibration can be continuously updated and corrected and consequently is robust to camera motion or sensor drift. The approach is demonstrated and evaluated using Azure Kinect cameras [1], although the proposed approach may be implemented using other commercial RGB and RGB-D sensors, as long as a 3D human pose estimation algorithm allows the reliable extraction and tracking of the human motions. Code and instructions will be made available to the public upon acceptance of the article.

The structure of this paper is as follows: Section 2 gives an overview of related work. Section 3 describes the proposed method. The evaluation results based on the Azure Kinect cameras are presented in Section 4, and the paper concludes with a discussion in Section 5.

## 2. Related Work

In this article, we chose to focus on the consumer level rather than high-end cameras, allowing the use of our code by the general public. Furthermore, we consider cameras that capture human body poses using non-intrusive technology (i.e., do not require wearable sensors or body markers), which is a major advantage. The “vicon” and “opto-track” system [5,6] are examples of SOTA technologies for performing 3D human pose estimation using multiple cameras. However, these high-end systems in addition to their high cost require a set of physical markers that must be worn by the human subject for pose capturing. These systems are impractical for use by the general public. Similarly, movement of the human body can be captured using inertial measurement unit (IMU) sensors [7], which are accurate, low cost and practical but are intrusive as they are attached to the body. Thus, several low-cost 3D cameras have recently been developed.

The Microsoft Azure Kinect [1] was released in March 2020, allowing developers and researchers easy access to a good quality RGB-D sensor. The Azure Kinect is widely compared against its predecessor the Kinect v2 in several recent studies [8,9,10]. Algorithms have also been developed for extracting the 3D human pose from RGB videos. Those include for example open pose [3] and mediapipe [11].

Multiple-camera setups allow increasing the coverage of the captured human body and minimizing occlusions. Multiple camera setups have been explored using various devices, such as RGB cameras [12], depth cameras, such as the Kinect V2 [13,14,15], and recently using Azure Kinect cameras [16].

Previous studies exploit 2D human poses in multiple 2D camera streams. Sinha and Pollefeys [17] presented a method for analyzing the motion of silhouettes in multiple video streams. Takahashi et al. [18] detect 2D human poses in videos for temporal synchronization, and in a recent study by Zhang et al. [19], synchronization is acheived by minimizing an energy function related to epipolar distances. These studies are based on 2D human pose data, which might be less accurate and require further processing in order to calculate the 3D pose estimation for pairs of cameras. Our proposed approach supports both RGB and RGB-D sensors, although RGB-D sensors may ensure that the 3D pose is captured with higher accuracy and in a wider range of environments compared to a standalone RGB sensor (e.g., low light conditions).

Our earlier studies rely on 3D human pose records, demonstrating a variant of the extrinsic calibration technique [13,14]. A recent study by Lee et al. [16] proposed the extrinsic calibration of multiple RGB-D cameras with 3D human pose estimation records and feature matching. Their proposed method refers only to the calibration process, where the temporal synchronization is achieved by connecting 3.5 mm audio cables between the devices. Our approach provides a complete end-to-end solution for both calibration and temporal synchronization, which in this paper is evaluated against the suggested methods of the sensor manufacturer both for calibration and temporal synchronization.

As discussed above, multiple camera setups require camera synchronization and calibration. Standard synchronization methods require coordination between the camera clocks so that concurrently recorded frames can be matched. This is usually performed by synchronizing the clocks on the host computers connected to the cameras using network-based clock synchronization methods (e.g., using an NTP server). Another approach is to physically connect the cameras using a cable. For instance, the method recommended by Microsoft for the Azure Kinect is to connect every two cameras with a dedicated 3.5 mm audio cable [20]; this will ensure all cameras capture synchronized frames (or with a constant predefined delay—see Section 3.1 and [20]).

Relying on clock synchronization methods introduces several major drawbacks:

- Requires external hardware (for example, connecting cables between cameras) which may limit the distance between cameras and limit the potential setup layout.
- Cannot be synchronized retrospectively; the clocks must be synchronized prior to the recording.
- Must be performed by a trained user who is familiar with the instructions of each synchronization method.

Our proposed approach offers an algorithm for synchronizing cameras which can be performed on-the-fly or even retrospectively based on asynchronous recordings of human body motions from the different views. It can be performed without any special training or dedicated hardware, and there are no limitations on the distances between cameras.

**Figure 2:**
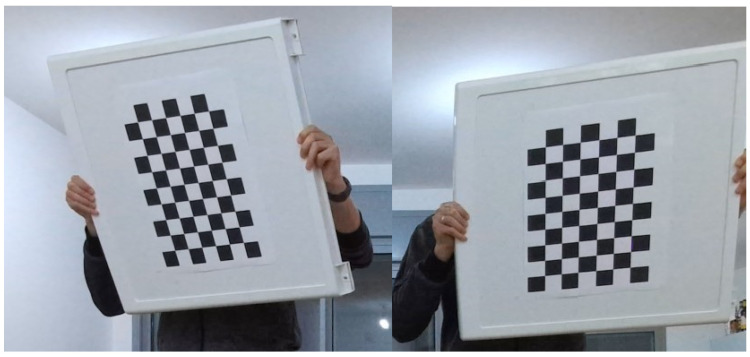
Checkerboard calibration: two corresponding frames from two synchronized camera streams.

Multi camera calibration typically requires dedicated calibration sessions preferably performed prior to recording. These methods require special accessories such as a flashlight [21] or checker board [22] (see Figure 2). Microsoft’s recommendation for calibrating multiple Azure Kinect is to use the checkerboard method [20]. Relying on such dedicated calibration sessions and tools introduces several major drawbacks:

- Requires a calibration session every time the sensor’s location changes.
- Cannot calibrate recordings retrospectively.
- Must be performed by a trained user who is familiar with the instructions of each calibration method.
- Typically does not support continuous calibration in real-time (on-the-fly).

Spatio-temporal calibration methods using multi-sensors records have been extensively researched in recent years. These methods aim to realize both the timing and the locations of the multi-camera setups simultaneously. A recent study [23] proposed a method for multiple RGB cameras which relies on the detection of multiple human bodies along videos and the calculation of spatio-temporal calibration from their locations and interactions. In another study [24], the authors proposed a spatio-temporal technique to synchronize and calibrate both an RGB camera and a 3D laser scanner, using the traditional chessboard as a target.

Our proposed approach offers an algorithm for calibrating the cameras that does not require specialized calibration sessions. It can be performed continuously on-the-fly during recording or even retrospectively. As with the synchronization, it relies only on the motion of a human body in the scene, whihc is captured from the different cameras. Thus, calibration can be continuously updated and corrected and consequently is robust to camera motion or sensor drift.

## 3. Method

**Figure 3:**
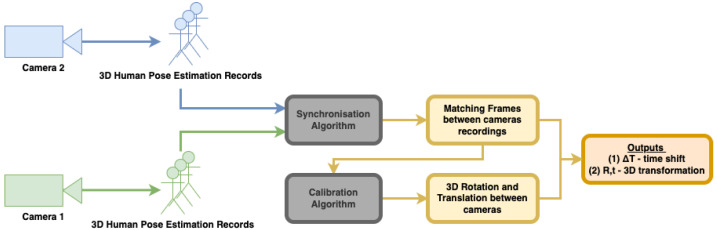
The workflow of the proposed spatio-temporal calibration approach.

In this section, we describe our approach to synchronization and calibration. A variant of the calibration technique was used in our earlier studies demonstrating its usefulness in a multi-camera system used to record patients within different medical studies [13,14,15]. In the current study, the algorithms are described and implemented on a Kinect Azure 2-camera setup; however, extensions to a larger number of cameras is straightforward. RGB sensors alone can be used rather than RGB-D, using 3D pose tracking algorithms [3,11]. As described above, the uniqueness of our approach is achieved by analyzing asynchronous 3D human pose estimation records instead of performing the traditional steps of synchronization and calibration of multiple cameras. Figure 3 shows the workflow of our proposed approach. It is important to note that although both algorithms for synchronization and calibration use the same body motion records, each of them analyzes the data differently. The synchronization algorithm uses body joint distances, whereas calibration uses joint positions.

### 3.1. Camera Synchronization

Using a 2-camera setup, we assume that two asynchronous streams of 3D human pose data are collected from the same scene. We assume the data of each camera is given as a body skeleton with 3D coordinates representing the skeleton joints (Figure 1). Synchronization is based on distances between body joints, that are, theoretically, invariant to camera pose. When a subject is in motion, the distances between body joints change. These changes should be consistent between camera views when the cameras are synchronized. Thus, monitoring the changes in the skeleton joint distances can serve the synchronization process.

**Figure 4:**
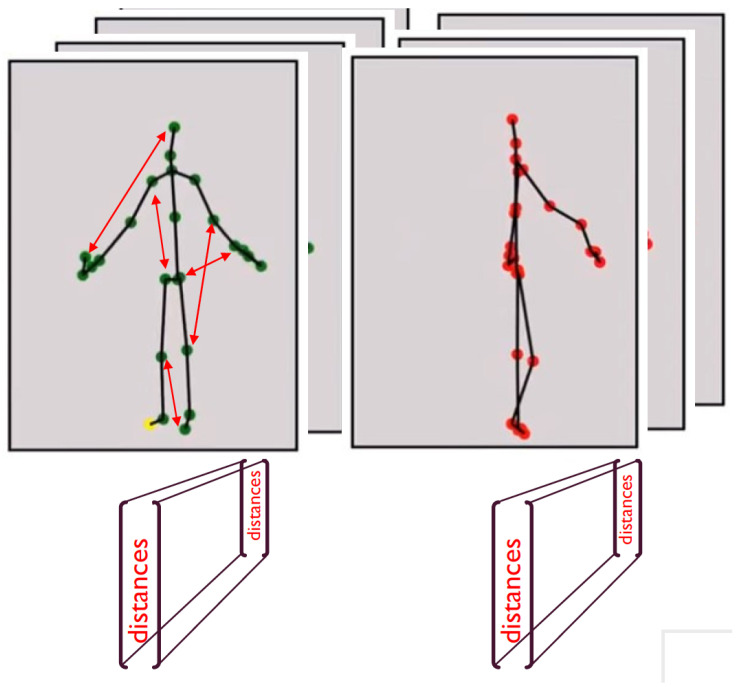
A vector (*PoseID*) of distance between every two skeleton joints is calculated for every frame of the input stream.

We define *PoseID* as the vector of (K×K)/2 distances between every 2 pairs of skeletal joints (with *K* being the number of joints in the skeleton). Thus, per input stream, we obtain a sequence of PoseIDs, one for each frame. Synchronization between cameras is performed by syncing the two PoseID streams (see Figure 4).

**Figure 5:**
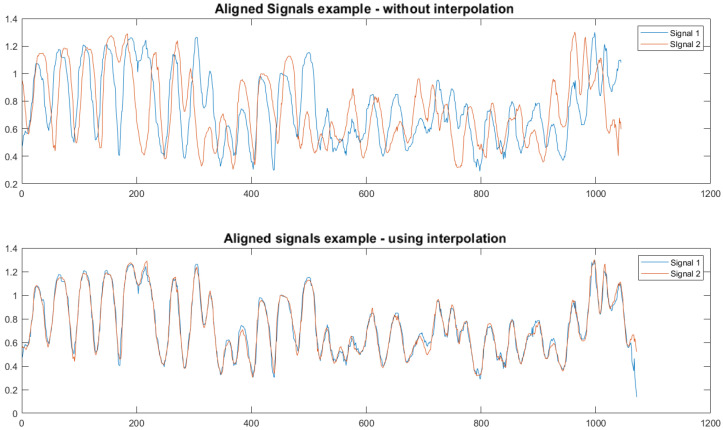
Examples of the aligned signals without (**top**) and with (**bottom**) re-sampling of the signals. Re-sampling allows overcoming the non-constant frame rate.

The two streams are not initially synchronized nor can we assume the same frame rate in both cameras nor a constant frame rate throughout the stream. Thus, to allow sub-frame rate synchronization, we re-sample the PoseID streams by interpolating the joint distances and increasing the frame rate synthetically. Specifically, we increase frame rate to 100 frames per second by using variable sampling between frames, according to each input stream’s time stamp (original frame rates in our tests ranged between 25 and 30). This improves synchronization significantly. Examples of synchronization results with and without re-sampling are shown in Figure 5.

Given the re-sampled sequences of PoseIDs from the two camera streams, we calculate the normalized cross-correlation [25,26] for each entry *i* in PoseID, and we take the coordinate of the peak value as the shift between streams with respect to the joint pair *i*. This results in (K×K)/2 peak correlation values and (K×K)/2 shift values. However, several of the joint pairs may be uninformative in that the distance between them does not change, such as consecutive joints on the skeleton (representing the ends of a skeleton “bone”) or body parts that do not move during the session. Thus, for every entry *i* in the PoseID sequences, its Coefficient of Variation ($CV_{i}$) [27] (Equation (1)) is computed over the correlation function $C_{i}$. A low CV value indicates an uninformative joint pair. To calculate the shift value between the two camera streams, the most informative joint pairs are selected, namely those associated with the top 10% CV value. The average of the shift values of these pairs is taken as the camera stream synchronization shift value.

(1) $$\hat{CV_{i}}=\frac{\mathrm{STD}(C_{i})}{\mathrm{MEAN}(C_{i})}$$

### 3.2. Camera Calibration

**Figure 6:**
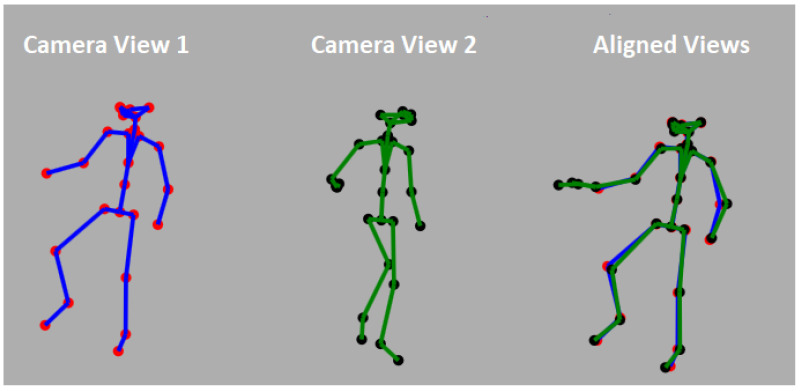
Skeleton alignment for two cameras positioned 180 degrees from each other.

As described above, in order to merge data from two or more cameras, a calibration procedure must be performed. That is, the transformation between the position of the two cameras must be computed and then used to align the recorded data. Rather than a specialized calibration session, we use an on-the-fly calibration method that is based only on the motion of a human body in the scene. In our system, a body skeleton (Figure 1) is computed per video frame, per camera. Every joint of the skeleton is associated with its 3D coordinate given in the camera’s frame of reference with XY axes parallel to the camera image plane and the Z-axis passing through the camera center. Every joint is also assigned a confidence value indicating the reliability of the 3D position as estimated by the tracking algorithm. Unfortunately, the skeleton joint positions are often erroneous due to errors in depth estimation or due to the occlusion of body parts from the camera’s line of sight. Unfortunately, the confidence values do not always reflect these errors. We further assume that the camera streams have been synchronized, and thus, we can assume matching skeletons between the cameras. The calibration transformation between cameras is given by the alignment between the skeletons obtained from the two camera streams. Figure 6 shows an example of a pair of aligned skeleton frames.

The Kabsch algorithm [28,29] is used to calculate the optimal transformation between cameras based on the skeleton joints. Assume two synchronized cameras A and B. Let *N* be the number of frames in the skeleton stream (of both cameras). For each frame *i* of camera A, define $P_{A}$ as the set of 3D coordinates of *k* skeleton joints. $P_{A}={\left\{p_{A}^{i}\right\}}_{i=1}^{k}$. Similarly, defined the set of skeleton joints for the corresponding frame in camera B: $P_{B}={\left\{p_{B}^{i}\right\}}_{i=1}^{k}$. The sets are normalized by subtracting their centroids, and their Cross-Covariance Matrix $H_{i}$ is calculated (Equation (2)). Singular Value Decomposition (SVD) is then applied on $H_{i}$ in order to calculate the 3D rotation $R_{i}$ and the translation vector $t_{i}$ between the skeletons of frame *i* (Equations (3)–(5)).

(2) $$H_{i}=\sum_{i=1}^{K}\left(p_{A}^{i}-{\mathrm{centroid}}_{A}\right){\left(p_{B}^{i}-{\mathrm{centroid}}_{B}\right)}^{T}$$

(3) $$\left[USV\right]=\mathrm{SVD}\left(H_{i}\right)$$

(4) $$R_{i}=VU^{T}$$

(5) $$t_{i}={\mathrm{centroid}}_{B}-R\times {\mathrm{centroid}}_{A}$$

This process is performed for each frame *i* of the skeleton streams. RANSAC [30] is then used to determine the optimal transformation between cameras. Thus, *k* skeleton joints are selected at a time to compute the transformation ($R_{i}$ and $t_{i}$), and the transformation with a maximal number of inlier joints is taken as the final calibration transformation. The *k* joints are pseudo-randomly selected by considering joints of high confidence originating from different parts of the skeleton (top, middle, and bottom parts of the skeleton). For additional details, see [13,14]. Figure 6 shows an example of two skeletons from two different views and the aligned results.

Further tuning and refinement of the calibration transformation can be obtained by considering the 3D cloud of points also recorded by the cameras. However, we found that this improvement is minor, and the calibration obtain using skeletons alone suffices in all our projects (see [13]).

It is important to note that our proposed algorithm also allows 180° setups (cameras facing each other), which is not possible to calibrate using the standard methods. The 180° setup can potentially provide coverage with a pair of cameras to generate a full 3D Body mesh.

## 4. Test Results

In this section, we report on the results of our tests, comparing our methods of synchronization and calibration against standard approaches. A data set of Kinect Azure recordings was collected to evaluate both methods. The synchronization and calibration methods use the same data set, which includes recordings in multiple setups, with different distances and angles between the sensors, and also by recording sessions with and without utilizing the ground truth techniques of both methods (see Section 4.1 and Section 4.2 for more details).

### 4.1. Synchronization Testing

Camera synchronization is typically performed by connecting between cameras in order to coordinate their internal clocks. One approach is to connect the two cameras into the same host computer and force frame captures to be coordinated with the same host computer clock. Another approach is to physically connect the cameras and trigger the camera captures simultaneously. Microsoft’s recommendation for synchronizing between Microsoft’s Azure Kinect sensors is to directly connect the cameras using a 3.5 mm audio cable [20]. Via the cable, the camera’s internal clocks are synchronized by forcing the cameras to start their recording session simultaneously. This approach has an inherent disadvantage in that it limits the user to the length of the audio cable. Additionally, increasing the number of cameras in a setup further complicates the synchronization. We compare our human motion-based synchronization method against Microsoft’s recommended technique, which we denote as *ground truth*.

**Figure 7:**
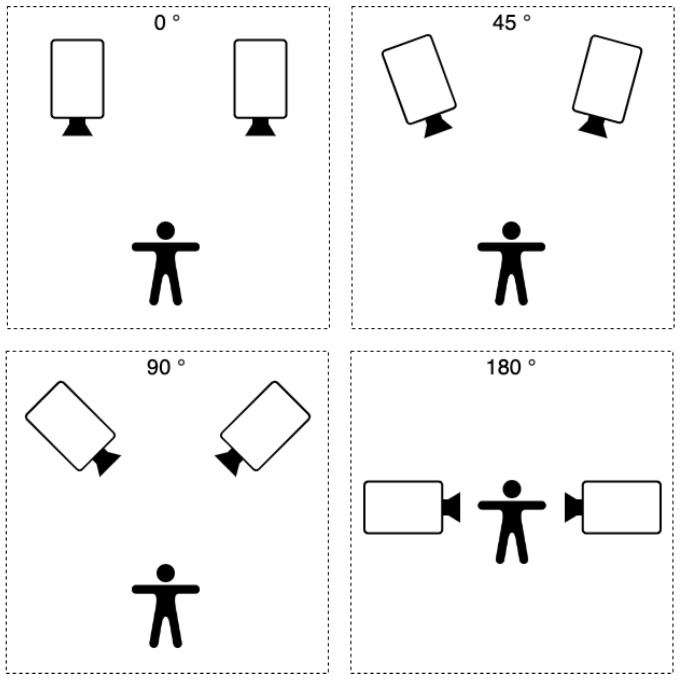
Multiple setups used for testing. Angles between cameras were: 0, 45, 90 and 180 rotation degree. Distances between cameras were 1 and 1.6 m.

To perform the comparison, we used two Microsoft Azure cameras positioned in 0, 30, 90 and 180 rotation degree to each other and a 1, 1.6 m distance between cameras, resulting in eight different testing setups (see Figure 7).

**Figure 8:**
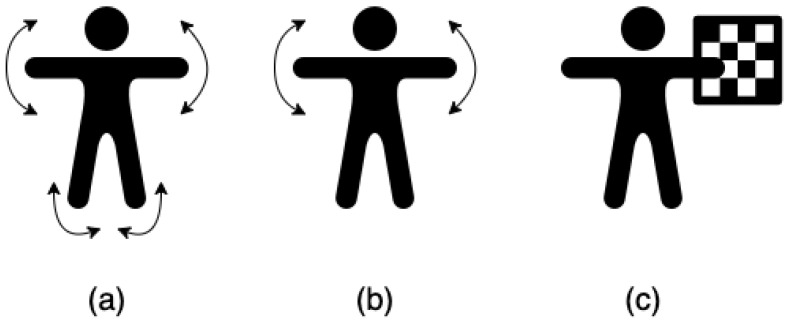
Three types of movements were recorded in each setup: (**a**) Moving all body parts, (**b**) Moving hands, and (**c**) Holding and moving a checkerboard.

For each setup, the two cameras recorded while connected with a 3.5 mm audio cable as per Microsoft’s instructions, as well as without the cable, so as to ensure inputs as per our proposed approach. Three types of sessions were recorded with respect to body motion (Figure 8): (1) the subject moved the entire body, (2) the subject moved his hands only, and (3) the subject held and waved a checkerboard without moving the rest of the body.

The Azure cameras collected the 3D body skeletons per frame together with a timestamp obtained from the internal clock of each camera. When the cameras were connected by the cable, the timestamps were synchronized and served as ground truth. The time shift in this case was 0.

**Figure 9:**
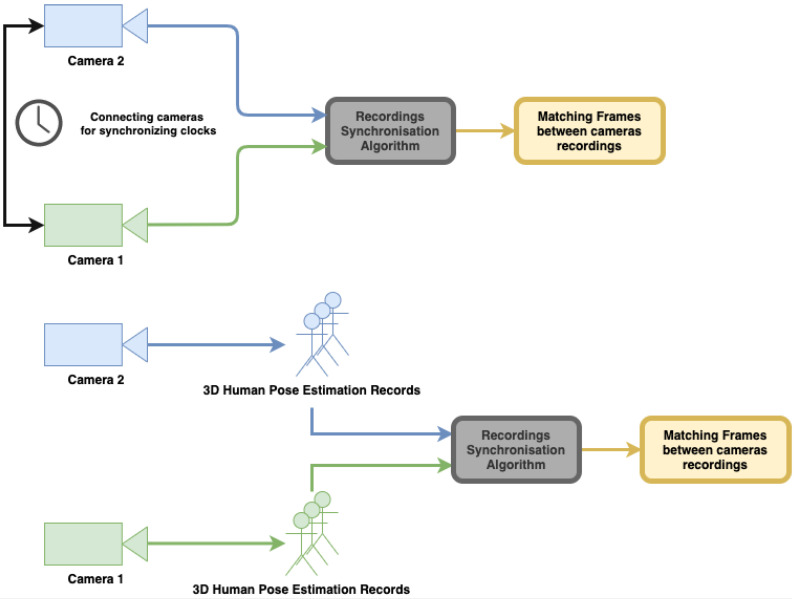
Comparing synchronization methods–Microsoft Azure method using a cable (**top**) vs. our human-motion based method (**bottom**).

In order to test our method, we applied the human motion based synchronization as described in Section 3.1 on every recorded session and compared the resulting shift value with the ground truth (where shift = 0) (see Figure 9).

Table 1 displays the results of the comparison tests between our proposed synchronization and the checkerboard method. Each row in Table 1 gives the shift error (in ms) between the two cameras for different camera setups and for the three different types of movements (moving the entire body, moving hands only and waving with a checkerboard). The bold values in Table 1 represent the movements with the lowest shift error for each tested setup. The ground truth synchronization is given by the cable based synchronization. Results show that our proposed synchronization method is accurate and robust across different inter-camera distances and rotation angles. The average shift error in all setups of moving the entire body and moving hands is 33.14 ms, and 31.85 ms, respectively, which are very similar, and both imply that our method produces outputs insignificantly different from the ground truth. The average shift error in all setups of holding a checkerboard is 110.66 ms, which is higher than other sessions, since most of the upper body is occluded by the checkerboard, and still, the error rate is relatively low. The checkerboard method cannot be implemented in setups of 180 deg; thus, the corresponding cell is empty in Table 1.

**Figure 10:**
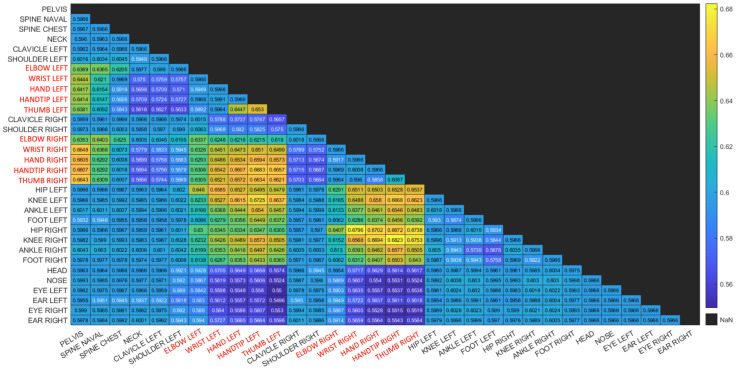
Matrix of Coefficient of Variation values (CV) for a session with hand movement only. It can be seen that the highest CV values are associated with at least one body joint from the hands (colored in red).

**Figure 11:**
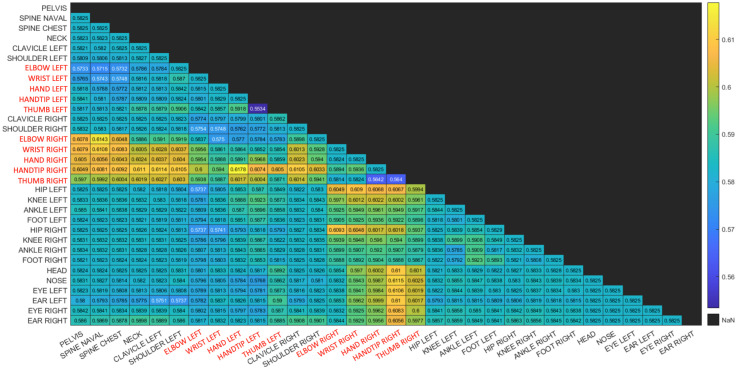
Matrix of Coefficient of Variation values (CV) for a session in which a checkerboard is waved. It can be seen that the highest CV values are associated with hand joints (colored in red) and face joints that are not occluded.

The results do not show any major difference between the three types of movements recorded, with the checkerboard session showing marginally higher values. This implies that the human-motion based synchronization is not sensitive to the type of movement the user is performing, as long as the entire body is tracked by both sensors and some basic movements are performed. Indeed, as described in Section 3.1, our proposed algorithm relies on the joint pairs with highest correlation coefficient. This is further demonstrated in Figure 10 and Figure 11 where matrices depict the Coefficient of Variance (CV) of the correlation function between pairs of joints. Matrix entries are color coded to enhance visualization. The body joints from the arms are colored in red.

The human motion-based calibration described above ranks joint pairs according to CV and considers joint pairs with high CV values as informative for synchronization. It can be seen in both matrices that high CV values appear for pairs of joints for which at least one of the joints is moving. Note that one moving joint and one constant joint is still informative for our proposed method. Figure 10 is a matrix of CV values for a session with hand movement only. It can be seen that the highest CV values are associated with at least one body joint from the hands. This is reasonable, as in this session, only the hands are moving. Figure 11 is a matrix of CV values for a session in which the subject is waving a checkerboard. Only some of the hand joints are associated with high CV values. This is due to the fact that the hands that are moving with the checkerboard are partially occluded (see Figure 2) and there is no other body motion. Note that although the position of the occluded hand joints that are moving is inferred, the low reliability of these joint positions is reflected in the low CV values and thus are not considered in the synchronization process. This is also reflected in the fact that different constant joints are selected within high-ranking joint pairs. Whereas in the hand motion sessions (Figure 10), joints such as the pelvis and spine joints are selected as constant joints, in the checkerboard session (Figure 11), face joints are selected, as they are more reliable than the occluded spine and pelvis joints.

These results display the flexibility and robustness of the synchronization algorithm, as it dynamically adapts to the recorded scene and selects the most reliable and informative skeleton joints for synchronization.

As another comparison, in [18], several synchronization methods were tested, resulting in errors in the range of 6 to 12 ms. Although a different experimental setup was used as well as different sensors, the presented errors are within the boundaries of our proposed method, and in part of our tested setups, our methods outperform them.

### 4.2. Multi-Camera Calibration Testing

Standard calibration methods require using special accessories such as the checkerboard calibration method [22] (see Figure 2).

**Figure 12:**
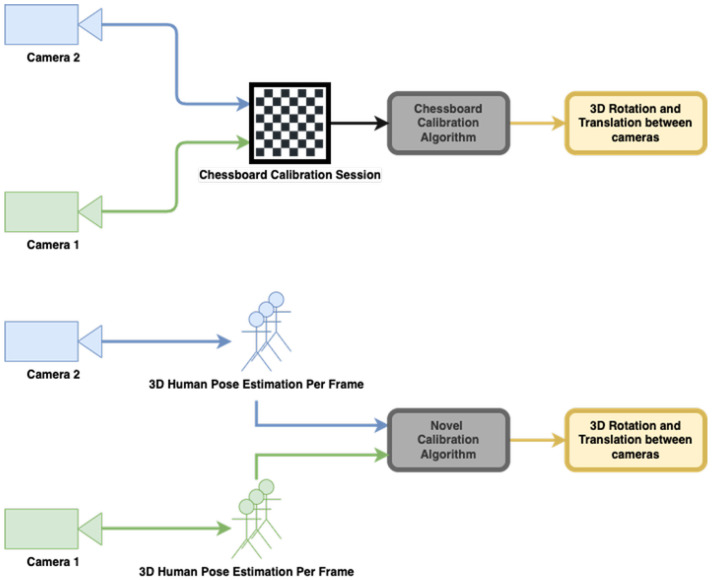
Comparing calibration methods: manual calibration using checkerboard (**top**) vs. our human-motion based calibration method (**bottom**).

In this section, we test our human-motion based calibration method and compare with the checkerboard method (Figure 12). As described in Section 3.2, our approach relies on the skeleton representation captured by the cameras and assumes the motion of the human subject in the recorded scene.

To test our human-motion based calibration algorithm, we tested the same 2-camera setups as used in the synchronization testing with a distance between cameras of 1 m and 1.6 m and varying degrees of rotations: 0°, 45°, 90° and 180° (Figure 7). Note that our proposed algorithm allows 180° setups (cameras facing each other), which is not possible to calibrate using the standard methods.

For each setup, both the checkerboard calibration and the human-motion based calibration were applied, resulting in two inter-camera transformations (each composed of a rotation matrix and a translation vector). For consistency and accurate comparisons, both the checkerboard and the body skeletons were acquired and/or transformed into the Kinect’s RGB camera coordinate system.

The following measurements were used to compare between the resulting transformations:

1. The mean rotation angle error is computed as the average difference between the Euler angles of the two rotation matrices.
2. The mean translation error is computed as the L2 difference between the two translation vectors.
3. The root mean square error (RMSE) is computed between a set of 3D points transformed by the checkerboard transformation and the same set of points transformed by the human motion-based transformation.

The three evaluation metrics were applied to the transformations computed in each of the tested setups excluding the 180° setup, in which the checkerboard method cannot be performed. Results are shown in Table 2. We further estimated the checkerboard calibration accuracy itself by repeating the calibration twice for each setup and evaluating the mean RMSE between pairs of resulting transformations. The checkerboard error for every setup is also given in Table 2. The human-based calibration method shows high accuracy when compared to the standard checkerboard method.

The mean rotation error and the mean translations error across all setups was found to be 0.44° and 2.46 cm, respectively. In a previous study [24], similar errors in computing the 3D transformation were reported with a 3D rotation error between 0.005° and 0.33° and translation error between 0.43 and 0.8 cm, implying similar results to our transformation errors. We note, however, that their experimental setups had the sensors much closer to each other, so their potential error is lower compared to the setups in our study.

The mean RMSE across all setups is 2.84 cm, which is similar to the checkerboard error. These results imply that the our proposed method is on par with the well-known checkerboard calibration method, with errors in the same range.

## 5. Conclusions

In this paper, we presented and tested a human-motion based synchronization and calibration methods. The proposed methods were shown to perform on par with standard methods and with the methods recommended by the camera’s manufacturer (Microsoft Kinect Azure).

The major advantage of our approach is that the calibration and synchronization methods do not require special equipment nor specialized calibration sessions and can be conducted on-the-fly. Furthermore, the system can automatically detect that camera positions have changed or that clock synchronization has been disrupted and re-calibrate. Thus, the cameras do not necessarily need to be steady, and the user can move the cameras between the sessions without requiring manual re-calibration.

Our proposed method could be used in various scenarios, such as modeling human body movement in order to better utilize exoskeletons (see recent study by Glowinski [7]). An interesting future study will be to replace the IMU sensors with our multiple cameras approach in order to model human body movement for exoskeletons, since our proposed method is easy-to-use compared to attaching IMU sensors to the captured human body, and also, our method will be able to deal with self body occlusions by using multi-camera setups.

Finally, the proposed approach promotes ease of use of multi-camera systems to be used by individuals not technically inclined. Basically, the human-motion-based synchronization and calibration allows for a set-up-and-go multi-camera system. Code and instructions will be made available to the public upon acceptance of the article.

## Figures and Tables

**Table 1:** Human motion-based synchronization results compared to Microsoft’s cable-based synchronization method.

Angle (deg.)	Distance (cm)	Moving Body Shift (ms)	Moving Hands Shift (ms)	Checkerboard Shift (ms)
0	100	**34**	59	106
45	100	18	**2**	99
90	100	24	**18**	62
0	160	**12**	62	31
45	160	**0**	**0**	11
90	160	143	**43**	355
180	300	**1**	39	-

**Table 2:** Human motion-based calibration is compared with the standard checkerboard calibration for each of the tested setups. Differences between the resulting transformations are given (see text). The measurement error of the checkerboard calibration method is also given.

Angle (deg.)	Dist. (cm)	Rota. Err. (deg.)	Transl. Err. (cm)	RMSE Err. (cm)	Checkerboard Err. (cm)
0	100	0.57	2.14	2.71	2.23
45	100	0.27	0.91	1.37	3.53
90	100	0.64	2.55	3.18	1.08
0	160	0.30	3.46	3.71	1.19
45	160	0.48	1.39	2.50	2.49
90	160	0.40	4.35	3.59	2.57
